# Inferring causal effects of homocysteine and B-vitamin concentrations on bone mineral density and fractures: Mendelian randomization analyses

**DOI:** 10.3389/fendo.2022.1037546

**Published:** 2022-11-28

**Authors:** Liwan Fu, Yuquan Wang, Yue-Qing Hu

**Affiliations:** ^1^ Center for Non-Communicable Disease Management, Beijing Children’s Hospital, Capital Medical University, National Center for Children’s Health, Beijing, China; ^2^ State Key Laboratory of Genetic Engineering, Human Phenome Institute, Institute of Biostatistics, School of Life Sciences, Fudan University, Shanghai, China; ^3^ Shanghai Center for Mathematical Sciences, Fudan University, Shanghai, China

**Keywords:** bone mineral density, homocysteine, B vitamins, fractures, Mendelian randomization

## Abstract

**Objectives:**

In the progress of bone metabolism, homocysteine (Hcy) and B vitamins play substantial roles. However, the causal associations of homocysteine, B-vitamin concentrations with bone mineral density (BMD), and fractures remain unclear. Therefore, we employed a two-sample Mendelian randomization (MR) design to infer the causal effects of Hcy and B vitamins on BMD and fractures.

**Methods:**

We selected instrumental variables from large genome-wide association studies (GWASs). Specifically, the exposures mainly included Hcy (sample size: 44,147), vitamin B12 (sample size: 45,576), folate (sample size: 37,465), and vitamin B6 (sample size: 1,864). The outcome variables included total body BMD (sample size: 66,628), heel BMD (sample size: 142,487), femoral neck BMD (sample size: 32,735), lumbar spine BMD (sample size: 28,498), and forearm BMD (sample size: 8143). Additionally, the total body BMD in several age strata was also included. Furthermore, the fractures of the forearm, femoral neck, lumbar spine, heel corresponding with the BMD regions, and femoral neck and lumbar spine BMD in men and women, separately, were added as additional outcomes. Two-sample MR approaches were utilized in this study. Inverse variance weighting (IVW) was adopted as the main analysis. MR-PRESSO, MR-Egger, the weighted median estimate, and multivariable MR were performed as sensitivity methods.

**Results:**

In the main analysis, Hcy concentrations have an inverse association with heel BMD (Beta = 0.046, 95% confidence interval (CI) -0.073 to -0.019, *P* = 9.59E-04) per SD unit. In addition, for one SD increase of vitamin B12, the total body BMD decreased 0.083 unit (95%CI -0.126 to -0.040, *P* = 1.65E-04). The trend was more obvious in age over 45 years (Beta = -0.135, 95%CI -0.203–0.067, *P* = 9.86E-05 for age 45-60; Beta = -0.074, 95%CI -0.141 to -0.007, *P* = 0.031 for age over 60 years). No association of B vitamins and Hcy levels with the risk of fractures and femoral neck and lumbar spine BMD in men and women was found in this study. Other sensitivity MR methods elucidated consistent results.

**Conclusions:**

Our findings indicated that there exist the inversely causal effects of Hcy and vitamin B12 on BMD in certain body sites and age strata. These give novel clues for intervening bone-related diseases in public health and nutrition.

## Introduction

The reduction of bone mineral density (BMD) and the deterioration of bone microarchitecture have been recognized as the primary characteristics of osteoporosis (1, 2). Osteoporosis is a chronic skeletal disorder, which contributes to the elevated risk of bone fragility and is susceptible to occurrence of osteoporotic fracture (1, 2). Osteoporosis is a common disease and has an increased global prevalence, becoming a main worldwide public health issue (3, 4). It was reported that over 30% of women and 20% of men beyond the age of 50 were affected by osteoporosis (5, 6). To date, utilizing the dual X-ray absorptiometry for gauging BMD becomes the current gold standard to diagnose osteoporosis (7). However, applying quantitative ultrasound for measuring BMD can provide additional information including bone size, geometry, and microarchitecture, which are poorly captured by dual X-ray absorptiometry (8). Notably, researchers proved that BMD is generally impacted by various risk factors, including gender (9), age (10), smoking (11), and alcohol consumption (12).

**Figure 1:**
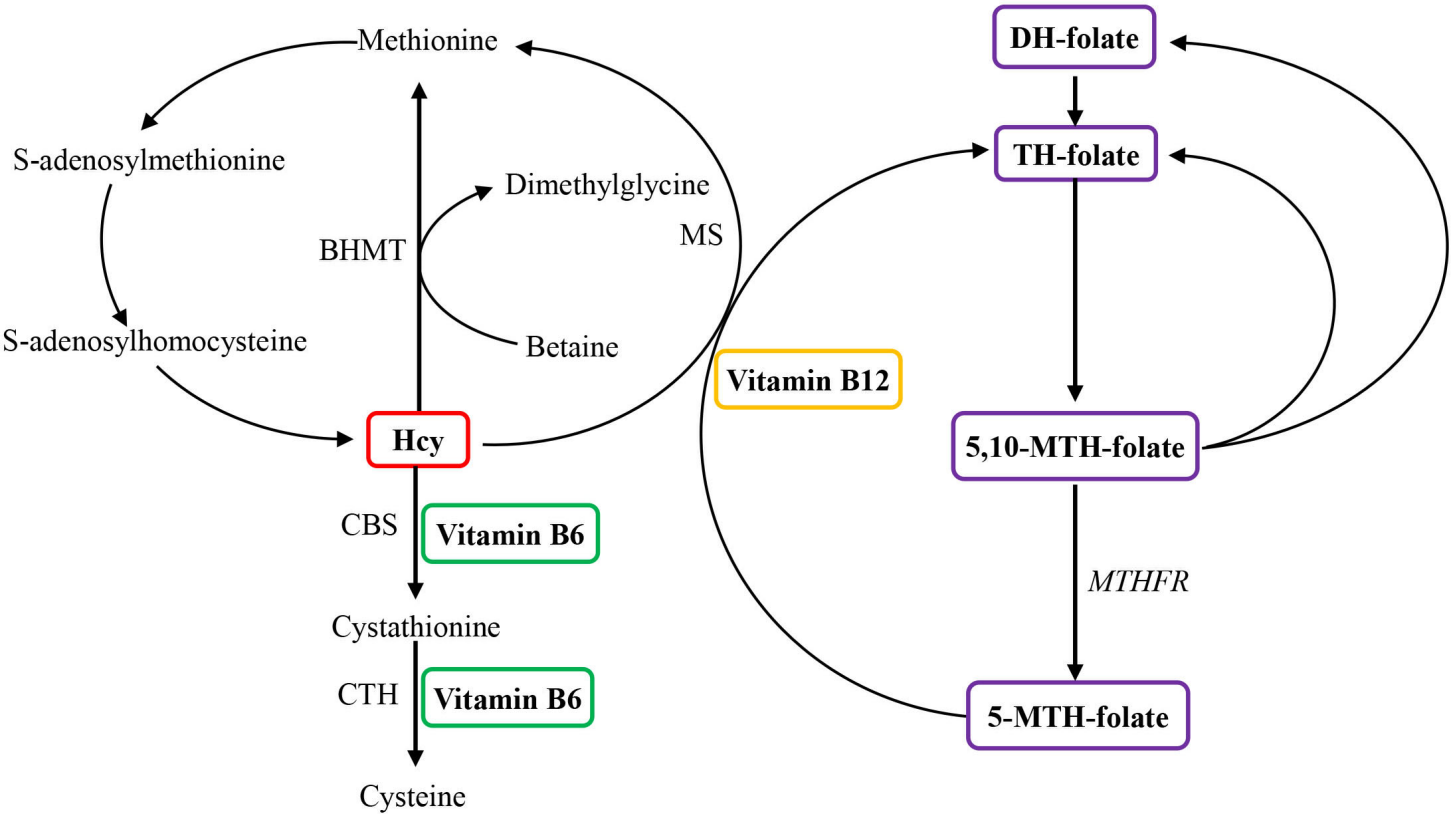
Overview of the role of vitamin B6, vitamin B12, and folate in homocysteine metabolism. Homocysteine is reconverted to methionine by acquiring a methyl group from 5-methyltetrahydrofolate, the positive form of betaine, or folate in the remethylation pathway. Irreversible removal of homocysteine happens across the transsulfuration pathway in which homocysteine condenses with serine to form cystathionine. BHMT, betaine homocysteine methyltransferase; CBS, cystathionine-β-synthase; CTH, cystathionine-gamma-ligase; DH, dihydo; Hcy, homocysteine; MS, methionine synthase (encoded by the MTR gene); MTH, methylenetetrahydrofolate; MTHFR, methylenetetrahydrofolate reductase; TH, tetrahydro.

In the general population, the lack of folate and vitamins B12 and B6 would result in elevated concentrations of homocysteine (Hcy) as these vitamins serve as cofactors for the varieties of enzymes involved in Hcy metabolism (**Figure 1**) (13, 14). The published studies indicated that milder elevation in Hcy levels was associated with a two- to fourfold elevated risk of hip and other fractures (15, 16). Some studies also revealed that increased Hcy concentrations might be associated with high osteoclast activity (17), elevated rates of bone turnover (17), and decreased BMD (18); some others have found no association of Hcy with BMD (19–21) and the markers of bone metabolism (22). A clinical trial utilizing randomized and placebo-controlled approaches implicated that folate and vitamin B12 concentrations over a 2-year period did not observe the changed levels of BMD or decreased fractures in elderly subjects with enhanced Hcy concentrations (23, 24). Thus, whether Hcy, together with B vitamins, directly influenced BMD and bone mass or is an “innocent bystander” is still kept unclear (25). It is necessary to take action to further investigate the relationship between BMD and the Hcy and B-vitamin concentrations.

The traditional observational studies have methodological limitations with the bias of undetected confounding factors and reverse causality, resulting in the deficiency of testing the causal associations between exposures and outcomes (26). Nowadays, the genetic variants, regarded as instrumental variables and being associated with exposures, were fully used by the Mendelian randomization (MR) design to act as the proxies of the risk factor for outcomes for inferring the causal effects of exposure and the outcome (27). Because of the genetic variants of offspring inherited randomly from their parents, in general, confounding factors are less likely to have an impact on these genetic instruments, and, hence, the studies with the MR design scarcely suffer from reverse causality and confounding factors (28).

**Figure 2:**
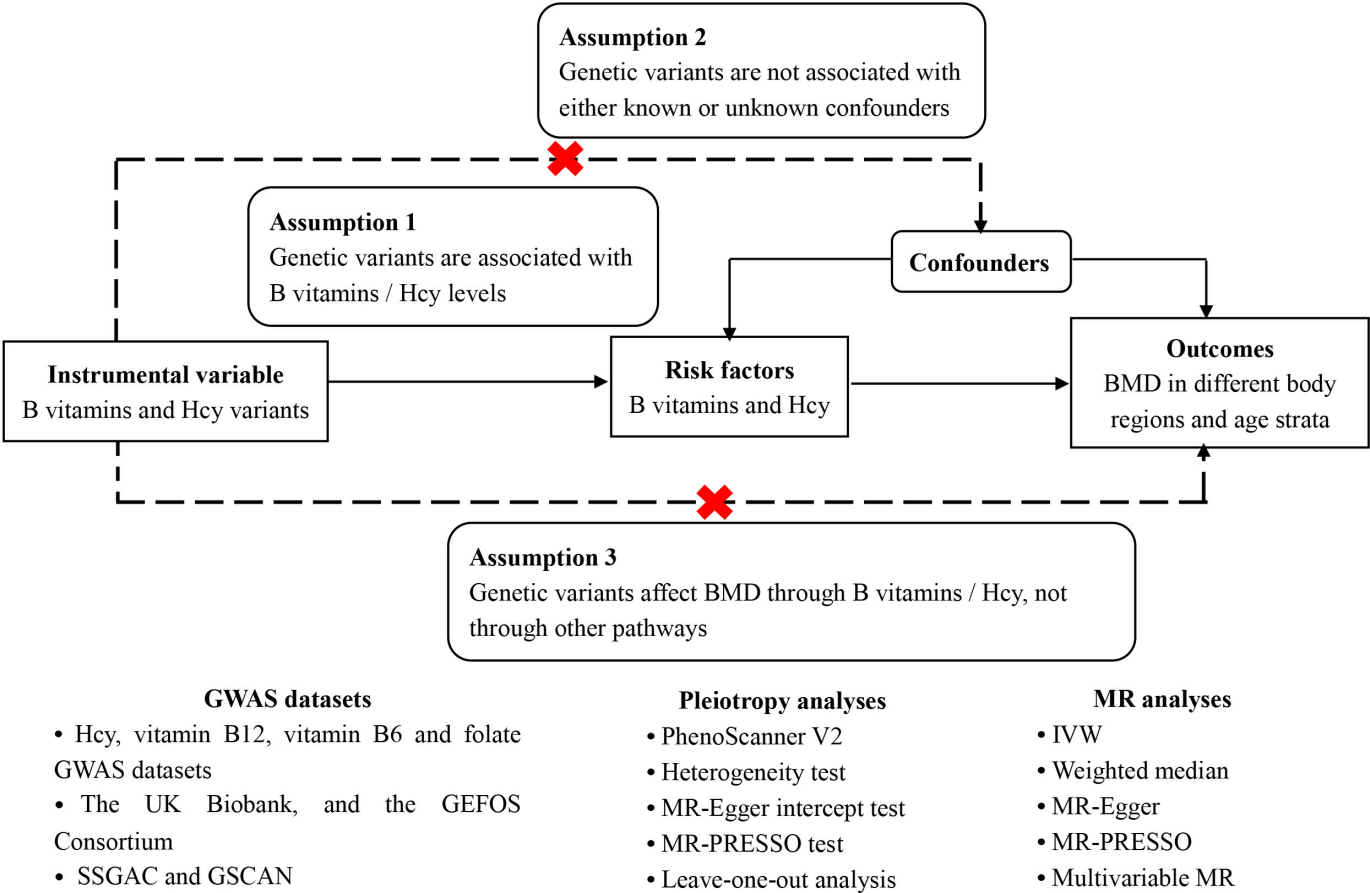
Schematic overview of the Mendelian randomization study design.

Given the abovementioned evidence that Hcy concentrations could probably lead to the changes of both bone mass and bone quality, together with the dependence of Hcy metabolism and B vitamins (29), as well as the controversial findings about the associations of B vitamins and Hcy with BMD in the observational studies (15, 19–21, 30–32), it is urgent to infer the causal associations of B vitamins and Hcy levels with BMD and then provide accurate causal effects. In this study, two-sample MR study approaches were carried out to explore the associations of the genetic prediction of B vitamins (folate and vitamins B12 and B6) and Hcy concentrations with total body BMD. Concurrently, different regions and age-specific associations between the concentrations of B vitamins, Hcy, and BMD were assessed for further exploration. The schematic overview of this MR study design is presented in **Figure 2**.

## Material and methods

We conducted a two-sample MR study using public GWAS data. Generally, MR should be satisfied with three conditions (**Figure 2**). First, as genetic instruments, the genetic variants have to be strongly associated with the exposures, which are B vitamins and Hcy in this study. We adopted the genome- wide significant level (*P* < 5E-08) together with r^2^ < 0.01 (this corresponds to linkage disequilibrium measure to define “independent genetic instrument”) as the inclusion criteria for genetic instruments. Second, no confounder exists in the relationships between the genetic instruments and the outcomes. Third is the exclusion restriction assumption (33, 34), namely, the genetic instruments affect the outcomes only through exposures. Horizontal pleiotropy testing could verify this assumption where the genetic instruments directly influence the outcomes or not (35).

### Ethical approval

Published or public data were used, and no original data were involved in this MR study. The original publications or included consortia described ethical approval and informed consent from every individual for the corresponding studies in the research.

### Selection of genetic instruments

**Figure 3:**
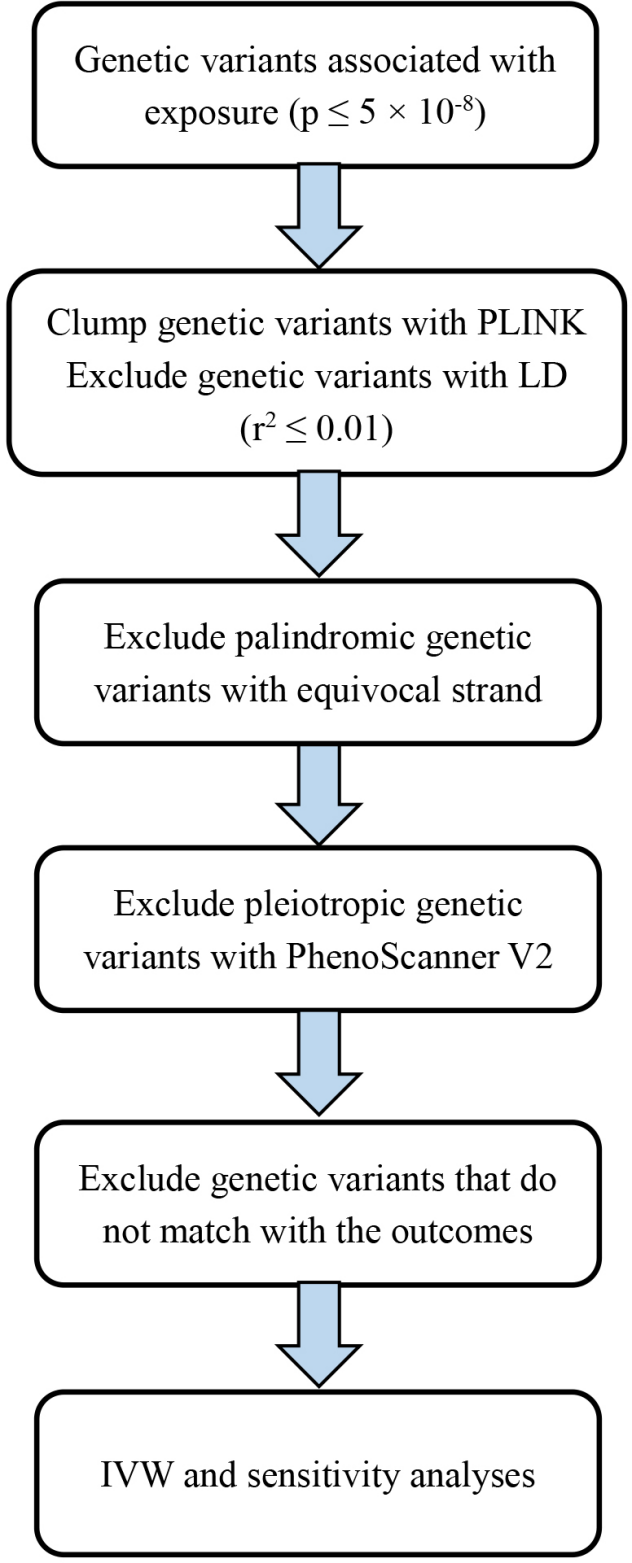
Flow chart of the Mendelian randomization study.

Based on the literature of exposures on serum vitamin B12 (sample size: 45,576) (36), blood Hcy (sample size: 44,147) (37), serum folate (sample size: 37,465) (36) and serum vitamin B6 (sample size: 1864) (38), SNPs that are associated with the corresponding exposures at the genome-wide significant level (*P* < 5E-08) were chosen as candidate genetic instruments, respectively. Then, we estimated the linkage disequilibrium among these SNPs for each exposure with the PLINK clumping method on the basis of 1000 Genome European reference panel as the GWASs are mostly from European origin. We also excluded palindromic variants with equivocal strands. Consequently, diverse SNPs without linkage disequilibrium (r^2^ < 0.01) were utilized as the genetic instruments (**Supplementary Table 1**). Specifically, there were 14 independent SNPs for Hcy, 14 independent SNPs for vitamin B12, 2 independent SNPs for folate, and 1 SNP for vitamin B6 (**Supplementary Table 1**). In total, the SNPs accounted for 6.0% of variance for Hcy (37) and vitamin B12 (36), respectively, and 1.3% of variance for vitamin B6 (38), as well as 1.0% of variance for folate (36). In case these SNPs for B vitamins and Hcy are associated with other correlated phenotypes, we used the internet resource (PhenoScanner V2) (39) and found that three SNPs (rs548987, rs42648, and rs838133) for Hcy and two SNPs (rs56077122 and rs34324219) for vitamin B12 were associated with other phenotypes (the search was conducted in April 2022), which might exert pleiotropic effects. Therefore, these five SNPs were removed. The flow chart of this MR study is shown in **Figure 3**. As the exposures, B vitamins and Hcy were transformed to one standard deviation (SD) unit. For testing the strength of genetic instruments, we calculated the *F*-statistics for different exposures (*F* = 256.1 for Hcy, *F* = 242.4 for vitamin B12, *F* = 189.2 for folate, and *F* = 24.5 for vitamin B6). All *F*-statistics were greater than 10, indicating the supportive evidence for the strength of genetic instruments.

### Datasets used for genetic association with outcomes

The forearm, femoral neck, lumbar spine, and heel are the prevalent sites of osteoporosis. The loss of BMD in these regions elevated the risk of osteoporosis and fractures compared to other body regions (40, 41). Thus, the BMD in these four body regions was firstly considered as outcomes. The summary statistics for BMD in the forearm, femoral neck, and lumbar spine were extracted from the GEnetic Factors for Osteoporosis (GEFOS) consortium (42), including 8,143, 32,735, and 28,498 subjects, respectively. For heel BMD, the summary statistics were provided by a GWAS dataset from UK Biobank (43), comprising 142,487 subjects. For BMD measured and validated across the study participants, heel BMD was done by quantitative ultrasound while other sites were done by dual X-ray absorptiometry.

We also explored the associations of B vitamins and Hcy with total body BMD (measured by dual X-ray absorptiometry) and BMD in different age strata (age strata referred to the total body BMD GWAS), which encompassed five age groups, namely, younger than 15 years old, 15–30, 30–45, 45–60, and older than 60 years old, as age has been deemed as a risk factor for becoming osteoporosis. According to a large GWAS meta-analysis (8), we acquired summary statistics for the total body BMD and BMD in these five age strata, which contained 66,628 subjects in total.

Before analyses in this MR study, all the outcomes of BMD were SD-transformed. A detailed information of data sources was displayed in **Table 1**.

**Table 1:** Descriptions of the genome-wide association studies (GWASs) employed in this Mendelian randomization study.

Exposure	Data source (PMID)	Sample size	%European	Covariates adjusted in research
Homocysteine (SD of log transformed)	PMID: 23824729	44,147	100	Age and sex and principal components in individual studies where applicable
Vitamin B12 (SD of quantile transformed)	PMID: 23754956	45,576	100	Age, year of birth, sex and the first principal component
Folate (SD of quantile transformed)		37,465		
Vitamin B6 (SD)	PMID: 19303062	1864	100	Not reported
**Outcomes**	**Data source (PMID)**	**Sample size**	**%European**	**Covariates adjusted in GWAS**
Forearm BMD (SD)	The GEFOS Consortium (26367794)	8,143	100	Age, age^2^, sex, and weight
Femoral neck BMD (SD)		32,735		
Lumbar spine BMD (SD)		28,498		
Heel BMD (SD)	The UK Biobank study (UKB, 28869591)	142,487	100	Genotyping array, age, sex, and the first four ancestry principal components
Total body BMD (SD)	The GEFOS Consortium (29304378)	66,628	86	Age, weight, height, and genomic principal components, as well as any additional study-specific covariates
Total body BMD of age 0–15 (SD)		11,807		
Total body BMD of age 15–30 (SD)		4,180		
Total body BMD of age 30–45 (SD)		10,062		
Total body BMD of age 45–60 (SD)		18,805		
Total body BMD of age over 60 (SD)		22,504		
**Confounders**	**Data source (PMID)**	**Sample size**	**%European**	**Covariates adjusted in research**
Years education attained (SD)	SSGAC (30038396)	766,345	100	Sex, birth year, their interaction, and 10 principal components of the genetic relatedness matrix
Smoking Heaviness (SD of cigarettes per day)	GSCAN (30643251)	337,334	100	Age, sex, age × sex interaction, and the first 10 genetic principle components
Alcohol (SD of log transformed drinks per week)		941,280		

BMD, bone mineral density; GEFOS, GEnetic Factors for OSteoporosis Consortium; GSCAN, GWAS and Sequencing Consortium of Alcohol and Nicotine use; SSGAC, Social Science Genetic Association Consortium; the summary-level data utilized in this study can be downloaded from the GWAS Catalog (https://www.ebi.ac.uk/gwas/) and Neale Lab (http://www.nealelab.is/uk-biobank).

Regarding the fact that fractures arising from fragile bones were clinically supposed to be a result of osteoporosis (44), the fractures of the forearm, femoral neck, lumbar spine, and heel corresponding with the BMD regions were added as additional outcomes. We extracted the summarized data for fractures from the Neale Lab (http://www.nealelab.is/uk-biobank), which encompassed 361,194 subjects, with 8,438 cases. Additionally, we explored the causal associations of Hcy and vitamin B12 with femoral neck and lumbar spine BMD in men and women, separately, for the consideration of the different effects because of sex. The data were from the GEFOS Consortium (45).

### Exploring possible sources of horizontal pleiotropy

Under the Instrument Strength is Independent of Direct Effect (InSIDE) assumption, the risk factors predicted by the genetic instruments should not influence both the exposures and outcomes simultaneously. In order to comply with this assumption, we assessed the possible relationship between the genetic instruments and risk factors, encompassing the degree of education (46), smoking, and alcohol using the rate (47). The descriptions of these variables are shown in **Table 1**.

### Harmonizing allele

In light of harmonizing the effect of alleles and their corresponding directions, we combined the genetic statistics from the GWAS of exposures and outcomes. The effect allele frequency was also utilized to make sure that palindromic genetic instruments were aligned properly. For SNPs that did not exist in the outcome datasets, we applied the substitute SNPs with r^2^ > 0.8 for the exposure-associated SNPs. Missing SNPs without matching substitutes for outcome GWAS summary statistics were ruled out from the subsequent analyses.

### Statistical analysis

Inverse variance weighting (IVW) with multiplicative random effects was employed as the main analysis (48). Additionally, four MR methods, including the weighted median estimate (49), MR-Egger (50), MR-PRESSO (51), and multivariable MR (52), were performed as sensitivity approaches for vitamin B12 and Hcy. IVW stands for the weighted regression slope of the effect of SNP-outcome on SNP-exposure assuming that the intercept is restricted to zero (48). When 50% SNPs are invalid instruments, the weighted median estimate could provide an unbiased evaluation (49). Under the InSIDE assumption, the horizontal pleiotropy can be estimated by the MR-Egger approach across the *P*-value for its intercept, as well as an assessment was given after the pleiotropic effects were adjusted. However, the MR-Egger approach may probably obtain wider confidence intervals (CIs) because of a lost statistical power (50). MR-PRESSO is another statistical method testing biases in the case of pleiotropy (the global test). It gives a corrected estimate *via* removing the outliers and supplies a distortion test, which evaluates whether the calculations with or without outliers obtain similar assessments under the InSIDE assumption (51). We also evaluated Cochran’s Q statistic to investigate the magnitude of heterogeneity (53) among the employed SNPs in every analysis. Subsequently, we utilized an online approach to calculate power (**Supplementary Table 4**) in addition to providing a supporting evidence of the strength of genetic instruments like *F*-statistics (54). Additionally, effects (betas) with the corresponding 95%CI were transformed to one SD increase in the genetic prediction of B vitamins and Hcy.

Linkage disequilibrium score regression (LDSC) was performed to assess sample overlap with LD hub (http://ldsc.broadinstitute.org/) (55) in case overlapping samples between two datasets bias the estimated causal effects. Considering the possibility that genetic instruments might have collider or ascertainment bias by conditioning on the possible confounders, we performed multivariable MR (52), which admits the direct effects of multiple variables on an outcome to be evaluated jointly. The effects of Hcy and vitamin B12 (because of insufficient instruments for folate and vitamin B6) on total body BMD and BMD in different body regions and age strata were assessed with adjusting for potential confounders, including educational attainments, smoking, and alcohol usage. We also conducted leave-one-out analysis to test the robustness of the significant main findings.

In terms of multiple testing adjustment, we employed a conservative *P*-value threshold of 1.25E-03 by Bonferroni correction, considering four exposures and 10 outcomes (0.05/40) for BMD. The *P*-value between the Bonferroni-adjusted significance level and the traditional significance level (*P* < 0.05) was regarded as suggestive significance. All analyses were performed by R Version 4.1.0 utilizing R packages (“TwoSampleMR”) (56), (“MRPRESSO”) (51), (“MendelianRandomization”) (57).

## Results

LDSC was employed to estimate overlapping samples between the exposure GWAS and the outcome GWAS. Results showed an approximately zero intercept of genetic covariance less than 10^-3^ in the pairs of the exposure–outcome GWAS (*P* > 0.1 by z-test in all pairs, data not shown), indicating approximately no sample overlap in the pairs of two datasets.

**Figure 4:**
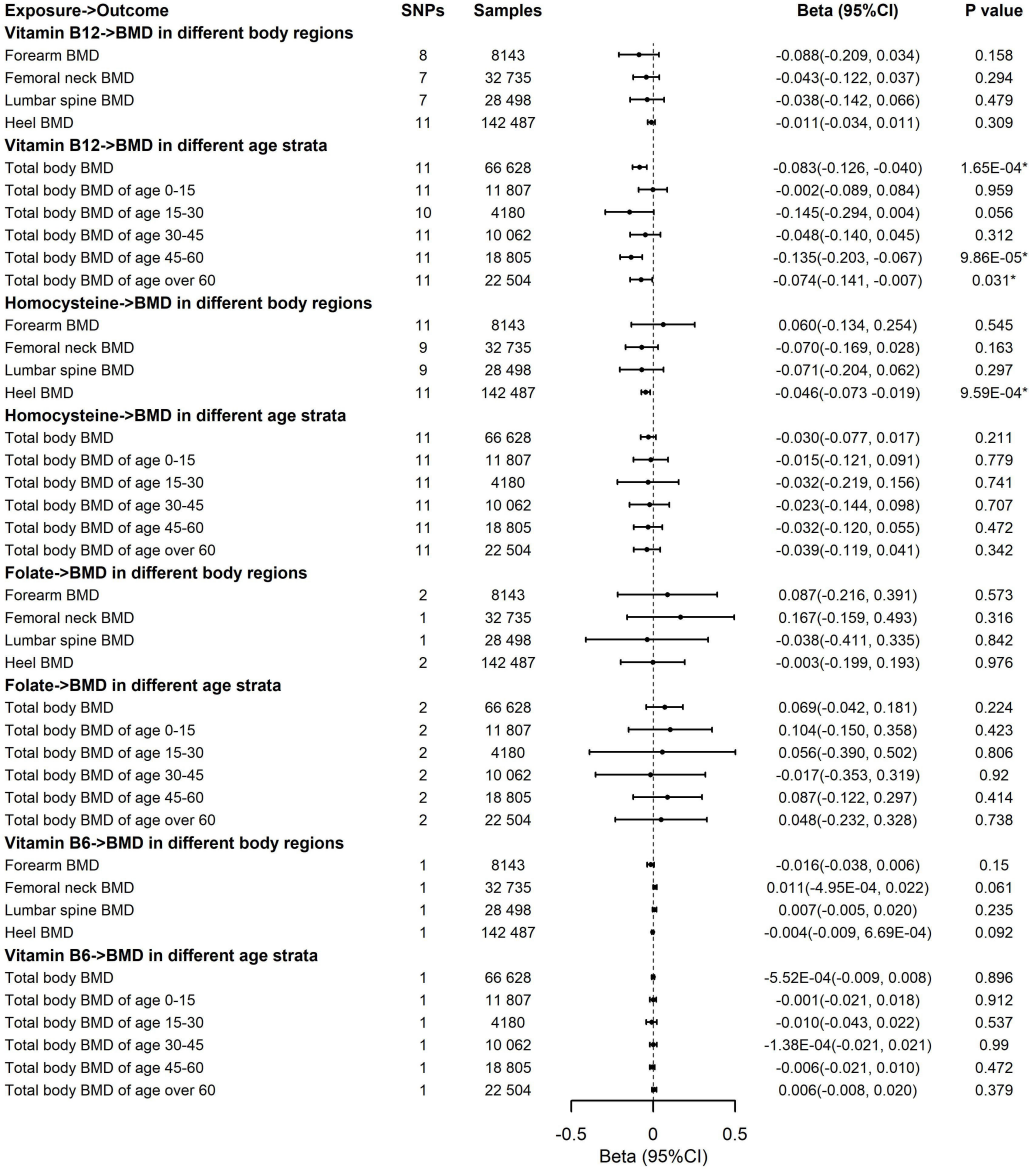
Effects of the genetic prediction of circulating vitamin B12, homocysteine, folate, and vitamin B6 on bone mineral density in different body regions and different age strata with the inverse variance weighting method. BMD, body mineral density; CI, confidence interval; GEFOS, GEnetic Factors for OSteoporosis Consortium; IVW, inverse variance weighting; The summary statistics data utilized in this study can be downloaded from the GWAS Catalog (https://www.ebi.ac.uk/gwas/) and Neale Lab (http://www.nealelab.is/uk-biobank); *The P-value reached the significant level; SNP, single-nucleotide polymorphism.

For the causal effects of B vitamins and Hcy on BMD in different body regions, we observed a significant association of the genetically predicted elevated concentrations of Hcy with decreased BMD in the heel region (**Figure 4**). For 1-SD increase in the genetic prediction of Hcy levels, the estimated beta was -0.046 (95CI, -0.073 to -0.019, *P* = 9.59E-04) for the 1-SD BMD of the heel. Results remained directionally consistent by the weighted median estimate and the MR-Egger and MR-PRESSO methods (**Supplementary Tables 5**, **7**). We noticed that there is no heterogeneity on the basis of Cochran’s Q statistic and no pleiotropy across the MR-Egger and MR-PRESSO methods for vitamin B12 and Hcy in the analyses of BMD in different body regions (**Supplementary Tables 5**–**7**). Leave-one-out analysis showed that the significant association between Hcy levels and heel BMD was not affected by single SNPs associated with Hcy concentrations (**Supplementary Figure 2**). The results were fairly robust from the outputs from various approaches. The genetic prediction of B-vitamin concentrations had no effect on BMD in different body regions (**Figure 4**). Multivariable MR analyses showed that the genetic prediction of increased Hcy levels was significantly associated with lower heel BMD (Beta, -0.041; 95%CI, -0.070 to -0.012; *P* = 6.28E-03) after adjusting for education attainment, smoking, and alcohol usage (**Supplementary Figure 1**). Thus, according to all sensitivity analyses, it is confident that the significance of the elevated concentrations of Hcy contributing to decreased heel BMD was credible in this study. In addition, we observed no association of B vitamins and Hcy levels with the risk of fractures of the four regions (**Supplementary Tables 8**–**10**) and no association of vitamin B12 and Hcy levels with femoral neck and lumbar spine BMD in men and women, separately (**Supplementary Tables 11**, **12**–**14**).

The associations of the genetic prediction of B vitamins and Hcy concentrations with total body BMD and BMD in different age strata are displayed in **Figure 4**. We observed the significant associations of genetically predicted elevated vitamin B12 with lower total body BMD (Beta, -0.083; 95%CI, -0.126 to -0.040; *P* = 1.65E-04), especially decreased total body BMD of age 45–60 (Beta, -0.135; 95%CI, -0.203 to -0.067; *P* = 9.86E-05) and age over 60 years (Beta, -0.074; 95%CI, -0.141 to -0.007; *P* = 0.031). No significant association was revealed in Hcy and other B vitamins with total body BMD in different age strata (**Figure 4**, **Supplementary Tables 5**, **7**). The significant findings of associations between vitamin B12 and total body BMD, as well as the BMD of age 45–60 and age over 60 kept consistent significantly by the weighted median estimate and the MR-PRESSO approach (**Supplementary Tables 6**, **7**). Additionally, Cochran’s Q statistic and the MR-Egger and MR-PRESSO approaches indicated no heterogeneity and no pleiotropy existing in these analyses (**Supplementary Tables 6**, **7**). In the multivariable MR analysis, the genetic prediction of increased vitamin B12 was more significantly associated with lower total body BMD (Beta, -0.085; 95%CI, -0.133, -0.038; *P* = 4.39E-04), total body BMD of age 45–60 (Beta, -0.128; 95%CI, -0.198, -0.058; *P* = 3.55E-04), and age over 60 years (Beta, -0.076; 95%CI, -0.145, -0.008; *P* = 0.029) after adjusting for education attainment, smoking, and alcohol usage (**Supplementary Figure 1**). Leave-one-out analysis showed that a single SNP was unable to influence the significant associations, confirming these significant results (**Supplementary Figure 2**).

Considering the confounding factors including education, smoking, and alcohol usage, we noticed that the effect alleles for the B vitamins and Hcy instruments (harmonized to increased concentrations of B vitamins and Hcy) were not associated with education, smoking, and alcohol usage (**Supplementary Table 3**), which suggested that the relationships between exposures and outcomes were unlikely to be influenced by these confounders.

## Discussion

In the present study, the genetic variants of exposures (SNPs-B vitamins and SNPs-Hcy) and outcomes (SNPs-BMD in different body regions and age strata) were collected from various large-scale GWAS data sources, and then, we conducted MR analyses to evaluate the causal effects of B vitamins and Hcy on the changes of total body BMD and BMD in distinct body sites and age strata. The findings of our study elucidated that the increased genetic prediction of Hcy concentrations was causally associated with the reduced heel BMD, and the elevated genetic prediction of vitamin B12 had a causal impact on decreased total body BMD, especially in the strata of age 45–60 and age over 60 years. Moreover, there was no association of B vitamins and Hcy concentrations with the risk of fracture of any of the bones and femoral neck and lumbar spine BMD in men and women.

Evidence showed that Hcy concentrations and B-vitamin levels had an effect on the change of BMD (15, 30–32), One study focused on elderly people disclosed the potential effect of Hcy on hip fracture, and its results therein indicated that the subjects with higher concentrations of Hcy had an increased risk of hip fracture than those with reduced Hcy levels, revealing that Hcy might be a vital factor for hip fracture in older persons (15). Therefore, it should be focused on age and fracture sites. The effects of Hcy levels on bone health may be different in distinct body regions and age strata. Another cross-sectional study has suggested the higher Hcy concentrations were inversely associated with the BMD in femoral neck and lumbar spine regions (58), which gave the support that different associations between Hcy levels and body regions for our motivation. Our findings declared that elevated Hcy levels had a significant influence on decreased heel BMD through the MR study design, revealing a novel clue for reducing heel BMD loss as heel BMD has been reported to be a vital risk factor for incident disability and mortality (59). Thus, this finding has great significance for public health.

With regard to the age-specific causal associations of B vitamins and Hcy with BMD, previous studies have demonstrated enhanced Hcy concentrations with decreased BMD in different ages (30, 60). A remarkable increase in Hcy concentrations with reduced BMD was found in postmenopausal women (60). Moreover, several scholars have explored the associations between Hcy and BMD in different ages, in which the concentrations of Hcy were negatively associated with the BMD of femoral neck, lumbar spine and hip regions in women aged under 50 (30). These indicated that age might be a risk for BMD loss. It would be better to test the effects of B vitamins and Hcy levels on BMD in different age strata. Our finding indicated that increased vitamin B12 significantly reduced BMD, especially in age over 45. Several observational studies unraveled that the elevated concentrations of vitamin B12 and folate have no effect on BMD (18, 20, 61–63). These negative results can be attributed to the multifactorial etiology of the disease and the different populations analyzed in these studies. The findings of the current study give relatively strong support for the significant causal association between vitamin B12 levels and total body BMD. Recently, an MR study by Wang et al. employed six SNPs associated with Hcy as instruments elucidated that genetically reduced Hcy was only associated with the increase of forearm BMD (64). However, too-few instruments would result in insufficient statistical power in this study by Wang et al. (64). In addition, some flaws existed in this study (64) as it elucidated that genetically reduced Hcy was associated with the increase of forearm BMD, while genetically determined Hcy elevation was not correlated with BMD. These are contradictory findings. The reasons of the inconsistent results were the effect alleles had to be unified before MR analysis was performed, and artificially dividing genetic instruments into two groups would reduce statistical power and lead to inaccurate results. Previously, we have developed genetic statistics to detect genetic variation in complex diseases and used MR approaches to investigate the causal relationships between complex diseases, including the casual association of B vitamins and Hcy with musculoskeletal diseases (28, 65–71). In the present study, we performed this MR using the latest instruments for Hcy and B-vitamin levels, offering both *F*-statistics and power calculations, to provide sufficient statistical power for inferring the causal effects of B vitamins and Hcy concentrations on total body BMD and BMD in different body regions and age strata. Compared to the study by Wang et al. (64), our study not only gave sufficient statistical power for inferring (effect alleles were unified before MR analysis) but also expanded the types of exposures (both Hcy and B vitamins) and outcomes (BMD in different body regions, sex and age strata, and fractures in different body regions). Furthermore, pleiotropy tests and sensitivity analyses (including multivariable MR) were also performed to ensure the robustness of our findings. Furthermore, Hcy and B vitamins were able to influence bone tissue formation by means of disrupting the development of collagen cross-links and reducing bone blood flow (72, 73). These support our findings that higher levels of Hcy and vitamin B12 mainly lead to bone catabolism.

Several mechanisms have been found to explain the relationships between BMD and B vitamins and Hcy levels. *In vitro* studies have demonstrated that Hcy and B vitamins could improve the activity and differentiation of osteoclasts and then induce the apoptosis of human bone marrow stromal cells *via* elevating reactive oxygen species (64). Furthermore, they also showed the ability of reducing bone blood flow, which was probably related to the mechanical bone properties (73). Specifically, Hcy and B vitamins produced apoptosis in bone marrow cells by means of the action of Nuclear Factor (NF)-kappa B and reactive oxygen species (74). The intracellular reactive oxygen species induced by them motivated osteoclast formation (75). Because the antioxidant N-acetyl cysteine could disrupt such negative impacts on bone cells (75), an enhancement in reactive oxygen species generated by them was able to have a substantial influence on the elevation in bone resorption in hyperhomocysteinemia. In addition, Hcy produced apoptosis in osteoblastic MC3T3-E1 cells by the virtue of inducing intracellular reactive oxygen species in a Hcy dose-dependent way (76). Age is a non-negligible risk factor for BMD. The average BMD may reduce considerably with age (77), particularly in subjects over 50 years. Dual X-ray absorptiometry and thoracic quantitative computed tomography suggested that bone mass reduces with age (78). Taken together, the above evidence revealed a possible pathogenic function of Hcy and B vitamins in the reduction of BMD, and age should be considered for further study.

The strengths of the present study came from the usage of MR, which could reduce residual confounding and diminish the possibility of false negatives by large GWASs. In addition, utilizing a series of non-overlapping data sources and applying more SNPs of B vitamins and Hcy were capable of enlarging sample size and explaining more phenotypic variance, respectively, as well as insuring statistical power. Notice that some participants present in the GWAS for BMD in distinct age strata were not restricted to European ancestry. This situation impeded the generalization of our results because the allele frequencies among populations were distinguished. Considering the likelihood of pleiotropy as an important issue in this study, we got directionally consistent results with the same significance in most sensitivity approaches (the weighted median estimate, MR-Egger, MR-PRESSO, and multivariable MR) after removing the obvious instruments with pleiotropy (**Supplementary Table 2**). The confounding factors encompassing education, smoking, and alcohol use were also tested in the case of confusing the relationships between exposures and outcomes since these confounders could influence BMD (11, 12). Moreover, no pleiotropic effect was observed in the findings of MR-Egger and MR-PRESSO, which suggested that unobserved pleiotropy and confounding did not bias our results. For the concern that vitamin B12 and Hcy may not be independent variables for causal inferring, actually, no matter vitamin B12 or Hcy, the three conditions (**Figure 2**) of conducting the MR study are satisfied. For avoiding the horizontal pleiotropy because of the interlink between vitamin B12 and Hcy, we used the strict screening criteria for selecting SNPs as instrumental variables. In other words, these instruments could absolutely infer the causal association between vitamin B12 and BMD and Hcy and BMD, respectively. However, our negative findings for vitamin B6 may reflect inadequate statistical power. Moreover, the present study provided little evidence on the association of B vitamins and Hcy levels with the risk of fracture. As one of the assumptions in MR is that the genetic variants affect outcomes only through exposure, the selected genetic variants for B vitamins and Hcy may not influence fractures *via* the Hcy metabolism. We only evaluate BMD in the forearm, femoral neck, lumbar spine and heel, which are common sites. Some BMD in other sites, such as BMD in spine, finger, rib, and scapula, would affect the total BMD. That is the reason why vitamin B12 showed association with total body BMD but not at any one of our evaluated sites. Moreover, dietary intake was not taken into account and the variation in each of the exposures that was explained by SNPs was small. Furthermore, both quantitative ultrasound and dual X-ray absorptiometry have their own advantages for evaluating BMD (7, 8). Due to the complex relationship between B vitamins and Hcy, as well as different measures for gauging BMD, further studies need to take the correlation between them into account to better assess their effects on BMD. Although we took into account the effects of age and sex on BMD, further studies should consider to explore data in separated populations according to the sex and menopausal status within the different gender groups (i.e., women >55 years old) since the hormonal status is the most important issue to consider.

## Conclusions

In conclusion, the present study demonstrated that the causalities of genetic prediction of Hcy increase with the reduced heel BMD, as well as genetic prediction of vitamin B12 elevation in relation to the total body BMD, especially in the strata of age 45–60 and age over 60, indicating that the concentrations of B vitamins and Hcy might play a crucial role in the development of bone health in distinct body regions and age strata. Future studies are, however, needed to explore the potential mechanisms by which B vitamins and Hcy regulate BMD. To this end, the levels of Hcy and vitamin B12 may be utilized in the early treatment of osteoporosis.

## Data availability statement

Publicly available datasets were analyzed in this study. The authors thank the UK Biobank study, and the summary statistics data of UKB can be download from Neale lab (http://www.nealelab.is/uk-biobank). The authors thank the GEFOS consortium, and the summary statistics data in GEFOS Consortium can be downloaded from the website (https://www.gefos.org/). The authors thank SSGAC for supplying the summary statistics of years of education attainment, which have been downloaded from https://www.thessgac.org/data. The authors thank GSCAN for supplying the summary statistics of smoking and alcohol use phenotypes, which have been downloaded from https://conservancy.umn.edu/handle/11299/201564.

## Author contributions

Study concept and design: LF and Y-QH; Acquisition of data: LF, Y-QH, and YW; Analysis and interpretation of data: LF; Drafting of the manuscript: LF and Y-QH; Critical revision of the manuscript for important intellectual content: LF, YW, and Y-QH; All authors have read and approved the final version of the manuscript.

## Funding

This study was supported by grants to LF and Y-QH from the National Natural Science Foundation of China (grants no. 82204063, 11971117, 11571082).

## Acknowledgments

The authors thank the UK Biobank study, and the summary statistics data of UKB can be downloaded from Neale lab (http://www.nealelab.is/uk-biobank). The authors thank the GEFOS consortium, and the summary statistics data in the GEFOS Consortium can be downloaded from the website (https://www.gefos.org/). The authors thank SSGAC for supplying the summary statistics of the years of educational attainment, which have been downloaded from https://www.thessgac.org/home. The authors thank GSCAN for supplying the summary statistics of smoking and alcohol use phenotypes, which have been downloaded from https://conservancy.umn.edu/handle/11299/201564. The authors thank all the reviewers and editors for their useful suggestions for improving this study.

## Conflict of interest

The authors declare that the research was conducted in the absence of any commercial or financial relationships that could be construed as a potential conflict of interest.

## Publisher’s note

All claims expressed in this article are solely those of the authors and do not necessarily represent those of their affiliated organizations, or those of the publisher, the editors and the reviewers. Any product that may be evaluated in this article, or claim that may be made by its manufacturer, is not guaranteed or endorsed by the publisher.
