# Supplementary material for: Inferring causal effects of homocysteine and B-vitamin concentrations on bone mineral density and fractures: Mendelian randomization analyses
Source: Front Endocrinol (Lausanne). 2022 Nov 28;13:1037546. doi: 10.3389/fendo.2022.1037546 (PMC9742470; doi:10.3389/fendo.2022.1037546)

Supplementary Material

**Supplementary Table 1.** Descriptions of instrumental variables

**Supplementary Table 2.** Pleiotropic examinations with applied SNPs for B vitamins and homocysteine

**Supplementary Table 3.** Genetic association of B vitamins, homocysteine instruments with confounders

**Supplementary Table 4.** Power evaluation

**Supplementary Table 5.** Associations of genetic prediction of circulating homocysteine with bone mineral density in different regions and age strata in sensitivity analyses.

**Supplementary Table 6.** Associations of genetic prediction of circulating vitamin B12 with bone mineral density in different body regions and age strata in sensitivity analyses

**Supplementary Table 7.** Associations of genetic prediction of circulating homocysteine and vitamin B12 with bone mineral density in different regions and age strata in the MR-PRESSO analysis

**Supplementary Table 8.** Associations of genetic prediction of B vitamins, homocysteine with fractures in different regions by IVW method.

**Supplementary Table 9.** Associations of genetic prediction of vitamin B12, homocysteine with fractures in different regions by MR-Egger and Weighted median estimate.

**Supplementary Table 10.** Associations of genetic prediction of vitamin B12, homocysteine with fractures in different regions by MR-PRESSO analysis

**Supplementary Table 11.** Associations of genetic prediction of vitamin B12, homocysteine with femoral neck BMD in men by various MR analyses

**Supplementary Table 12.** Associations of genetic prediction of vitamin B12, homocysteine with femoral neck BMD in women by various MR analyses

**Supplementary Table 13.** Associations of genetic prediction of vitamin B12, homocysteine with lumbar spine BMD in men by various MR analyses

**Supplementary Table 14.** Associations of genetic prediction of vitamin B12, homocysteine with lumbar spine BMD in women by various MR analyses

**Supplementary Figure 1.** Effects of genetic prediction of circulating vitamin B12 and homocysteine on BMD in different body regions and different age strata with the Multivariable Mendelian randomization adjusting education attained, smoking and alcohol usage

**Supplementary Figure 2.** Forest plot for leave-one-out analysis of the significant IVW results of homocysteine with heel BMD, Vitamin B12 with total body BMD, total body BMD of age 45-60 and age over 60, with each point denoting the causal effect by IVW after removing the specific SNP on the left side

**Supplementary Table 1.** Descriptions of instrumental variables

| SNP | Chr | Position | EA | OA | EAF | Effect | SE | *P* value | Mapped Gene | Exposure |
| --- | --- | --- | --- | --- | --- | --- | --- | --- | --- | --- |
| rs4654748 | 1 | 21786068 | T | C | 0.50 | 1.450 | 0.280 | 8.30E-18 | *ALPL* | Vitamin B6 |
| rs2270655 | 4 | 146576418 | G | C | 0.94 | 0.066 | 0.018 | 2.20E-13 | *MMAA* | Vitamin B12 |
| rs1141321 | 6 | 49412433 | C | T | 0.63 | 0.061 | 0.007 | 3.60E-26 | *MUT* | Vitamin B12 |
| rs7788053 | 7 | 86773722 | A | G | 0.25 | 0.046 | 0.007 | 1.70E-10 | *FUT6* | Vitamin B12 |
| rs1801222 | 10 | 17156151 | G | A | 0.59 | 0.110 | 0.007 | 3.30E-75 | *CUBN* | Vitamin B12 |
| rs56077122 | 10 | 17207015 | A | C | 0.34 | 0.087 | 0.009 | 4.80E-21 | *CUBN/TRDMT1* | Vitamin B12 |
| rs12272669 | 11 | 71392610 | A | G | 0.01 | 0.510 | 0.007 | 3.00E-09 | *MMACHC* | Vitamin B12 |
| rs34324219 | 11 | 59623378 | C | A | 0.88 | 0.210 | 0.007 | 1.10E-111 | *TCN1* | Vitamin B12 |
| rs34528912 | 11 | 59631535 | T | C | 0.04 | 0.170 | 0.021 | 2.10E-15 | *TCN1* | Vitamin B12 |
| rs117456053 | 11 | 59616831 | G | A | 0.98 | 0.160 | 0.026 | 1.90E-09 | *TCN1* | Vitamin B12 |
| rs41281112 | 13 | 100518634 | C | T | 0.95 | 0.170 | 0.020 | 8.90E-35 | *CLYBL* | Vitamin B12 |
| rs3742801 | 14 | 74759006 | T | C | 0.29 | 0.045 | 0.009 | 1.70E-13 | *ABCD4* | Vitamin B12 |
| rs2336573 | 19 | 8367709 | T | C | 0.03 | 0.320 | 0.007 | 8.40E-59 | *CD320* | Vitamin B12 |
| rs602662 | 19 | 49206985 | A | G | 0.60 | 0.160 | 0.007 | 2.40E-139 | *FUT2* | Vitamin B12 |
| rs1131603 | 22 | 31018975 | C | T | 0.06 | 0.190 | 0.017 | 4.90E-49 | *TCN2* | Vitamin B12 |
| rs1801133 | 1 | 11856378 | G | A | 0.67 | 0.096 | 0.008 | 9.50E-53 | *MTHFR* | Folate |
| rs652197 | 11 | 71849741 | C | T | 0.18 | 0.069 | 0.011 | 1.40E-12 | *FOLR3* | Folate |
| rs1801133 | 1 | 11856378 | A | G | 0.34 | 0.158 | 0.007 | 4.30E-104 | *MTHFR* | Homocysteine |
| rs2275565 | 1 | 237048676 | G | T | 0.79 | 0.054 | 0.009 | 2.00E-10 | *MTR* | Homocysteine |
| rs4660306 | 1 | 45978675 | T | C | 0.33 | 0.043 | 0.007 | 2.30E-09 | *MMACHC* | Homocysteine |
| rs1047891 | 2 | 211540507 | A | C | 0.33 | 0.086 | 0.008 | 4.60E-27 | *CPS1* | Homocysteine |
| rs9369898 | 6 | 49382193 | A | G | 0.62 | 0.045 | 0.007 | 2.20E-10 | *MUT* | Homocysteine |
| rs548987 | 6 | 25869371 | C | G | 0.13 | 0.060 | 0.010 | 1.10E-08 | *SLC17A3* | Homocysteine |
| rs42648 | 7 | 89977760 | G | A | 0.60 | 0.039 | 0.007 | 2.00E-08 | *GTPB10* | Homocysteine |
| rs1801222 | 10 | 17156151 | A | G | 0.34 | 0.045 | 0.007 | 8.40E-10 | *CUBN* | Homocysteine |
| rs12780845 | 10 | 17223244 | A | G | 0.65 | 0.053 | 0.009 | 7.80E-10 | *CUBN* | Homocysteine |
| rs7130284 | 11 | 89148372 | C | T | 0.93 | 0.124 | 0.013 | 1.90E-20 | *NOX4* | Homocysteine |
| rs2251468 | 12 | 121405126 | C | A | 0.35 | 0.051 | 0.007 | 1.30E-12 | *HNF1A* | Homocysteine |
| rs154657 | 16 | 89708096 | A | G | 0.47 | 0.096 | 0.007 | 1.70E-43 | *DPEP1* | Homocysteine |
| rs838133 | 19 | 49259529 | A | G | 0.45 | 0.042 | 0.007 | 7.50E-09 | *FUT2* | Homocysteine |
| rs234709 | 21 | 44486964 | C | T | 0.55 | 0.072 | 0.007 | 3.90E-24 | *CBS* | Homocysteine |

Chr, chromosome; EA, effect allele; EAF, effect allele frequency; OA, other allele; SE, standard error; SNP, single nucleotide polymorphism.

**Supplementary Table 2.** Pleiotropic examinations by applying SNPs for B vitamins and homocysteine

| Exposure/SNP | Mapped Gene | Effect allele | Phenotypes | Direction |
| --- | --- | --- | --- | --- |
| Vitamin B6 | | | | |
| rs4654748 | *ALPL* | T | Alkaline phosphatase | - |
| Vitamin B12 | | | | |
| rs2270655 | *MMAA* | G | NA |  |
| rs1141321 | *MUT* | C | NA |  |
| rs7788053 | *FUT6* | A | NA |  |
| rs1801222 | *CUBN* | G | NA |  |
| rs56077122 | *CUBN/TRDMT1* | A | Platelet distribution width | + |
|  |  |  | Female genital prolapse | + |
|  |  |  | Mean corpuscular volume | - |
|  |  |  | Disorders of refraction and accommodation | + |
|  |  |  | Self-reported dupuytrens contracture | - |
|  |  |  | Plateletcrit | - |
| rs12272669 | *MMACHC* | A | NA |  |
| rs34324219 | *TCN1* | C | Self-reported pernicious anaemia | - |
|  |  |  | Cause of death: hodgkins disease, unspecified | + |
|  |  |  | ischemic stroke | + |
| rs34528912 | *TCN1* | T | NA |  |
| rs117456053 | *TCN1* | G | NA |  |
| rs41281112 | *CLYBL* | C | NA |  |
| rs3742801 | *ABCD4* | T | NA |  |
| rs2336573 | *CD320* | T | NA |  |
| rs602662 | *FUT2* | A | Vitamin B12 female | + |
|  |  |  | Circulating Vitamin B12 concentration in total cholesterol | + |
|  |  |  | Folate pathway vitamin levels | + |
|  |  |  | Vitamin B12 | + |
| rs1131603 | *TCN2* | C | NA |  |
| Folate | | | | |
| rs1801133 | *MTHFR* | G | Diastolic blood pressure | - |
|  |  |  | Mean corpuscular hemoglobin | - |
| rs652197 | *FOLR3* | C | NA |  |
| Homocysteine | | | | |
| rs1801133 | *MTHFR* | A | Diastolic blood pressure | - |
|  |  |  | Mean corpuscular hemoglobin | - |
| rs2275565 | *MTR* | G | NA |  |
| rs4660306 | *MMACHC* | T | NA |  |
| rs1047891 | *CPS1* | A | Homocysteine levels | + |
|  |  |  | Homocysteine in fat mass | + |
|  |  |  | Basal metabolic rate | + |
|  |  |  | Platelet count | + \| - |
| rs9369898 | *MUT* | A | NA |  |
| rs548987 | *SLC17A3* | C | Mean corpuscular hemoglobin | - |
|  |  |  | Red cell distribution width | + |
|  |  |  | Hemoglobin concentration | - |
|  |  |  | Primary sclerosing cholangitis | + |
|  |  |  | Mean corpuscular volume | - |
|  |  |  | Hematocrit | - |
|  |  |  | Intestinal malabsorption | + |
|  |  |  | Reticulocyte count | - |
|  |  |  | IgA deficiency | + |
|  |  |  | Schizophrenia | - |
|  |  |  | Lymphocyte count | - |
|  |  |  | Forced expiratory volume | - |
|  |  |  | Leg fat mass | + |
|  |  |  | Monocyte count | - |
|  |  |  | Self-reported gout | + |
|  |  |  | Serum urate | + |
|  |  |  | White blood cell count | - |
|  |  |  | Self-reported sarcoidosis | + |
|  |  |  | Body mass index | + |
|  |  |  | Headache | - |
| rs42648 | *GTPB10* | G | Impedance of arm right | - |
|  |  |  | Impedance of whole body | - |
|  |  |  | Arm predicted mass right | + |
| rs1801222 | *CUBN* | A | NA |  |
| rs12780845 | *CUBN* | A | NA |  |
| rs7130284 | *NOX4* | C | NA |  |
| rs2251468 | *HNF1A* | C | Homocysteine levels | + |
|  |  |  | Homocysteine levels in coronary artery disease | - |
|  |  |  | Homocysteine in total cholesterol | + |
|  |  |  | Gamma glutamyl transferase | - |
|  |  |  | C-reactive protein | + |
| rs154657 | *DPEP1* | A | Self-reported hypertension | - |
|  |  |  | Mean corpuscular volume | + |
|  |  |  | Hematocrit | + |
|  |  |  | log eGFR creatinine in non-diabetics | - |
| rs838133 | *FUT2* | A | Sodium in urine | - |
|  |  |  | Mean platelet volume | - |
|  |  |  | Hip circumference | - |
|  |  |  | Sitting height | - |
|  |  |  | Percentage of total caloric intake from macronutrients protein | Not reported |
|  |  |  | Dietary macronutrient intake | - |
|  |  |  | Total cholesterol | + |
|  |  |  | Cholelithiasis | + |
| rs234709 | *CBS* | C | Blood and toenail selenium levels | Not reported |

SNPs in red color means pleiotropy; NA, not available; SNP, single nucleotide polymorphism. These associations were identified at the genome-wide significance level from the PhenoScanner V2, a database of human genotype-phenotype associations (http://www.phenoscanner.medschl.cam.ac.uk/).

**Supplementary Table 3.** Genetic association of B vitamins, homocysteine instruments with confounders

| Exposure | Confounders | Effect | SE | *P* value |
| --- | --- | --- | --- | --- |
| Vitamin B6 | Education (SD) | -1.10E-04 | 2.83E-04 | 0.685 |
|  | Alcohol (SD of log transformed drinks per week) | -9.60E-04 | 1.35E-03 | 0.476 |
|  | Tobacco (SD of cigarettes per week) | 5.82E-03 | 3.98E-03 | 0.144 |
| Vitamin B12 | Education (SD) | 1.54E-03 | 1.69E-03 | 0.361 |
|  | Alcohol (SD of log transformed drinks per week) | 0.013 | 0.012 | 0.254 |
|  | Tobacco (SD of cigarettes per week) | -0.022 | 0.014 | 0.121 |
| Folate | Education (SD) | 6.73E-03 | 0.004 | 0.095 |
|  | Alcohol (SD of log transformed drinks per week) | -0.025 | 0.019 | 0.196 |
|  | Tobacco (SD of cigarettes per week) | 0.061 | 0.054 | 0.255 |
| Homocysteine | Education (SD) | -2.96E-03 | 2.33E-03 | 0.204 |
|  | Alcohol (SD of log transformed drinks per week) | 2.56E-03 | 7.97E-03 | 0.748 |
|  | Tobacco (SD of cigarettes per week) | 0.061 | 0.044 | 0.171 |

Effects were obtained from the random-effects invariance weighted median model; SE, standard error.

**Supplementary Table 4.** Power evaluation

| Outcomes | Source | | Sample size | Beta_B6_ | Beta_B12_ | Beta_folate_ | Beta_Hcy_ | ~6% of variance  Beta at 80% power | | ~1% of variance  Beta at 80% power | |
| --- | --- | --- | --- | --- | --- | --- | --- | --- | --- | --- | --- |
|  |  |  |  |  |  |  |  | ≤ lower | ≥ upper | ≤ lower | ≥ upper |
| Forearm BMD | | GEFOS | 8143 | -0.016 | -0.088 | 0.087 | 0.060 | -0.13 | 0.13 | -0.33 | 0.33 |
| Femoral neck BMD | | GEFOS | 32,735 | 0.011 | -0.043 | 0.167 | -0.070 | -0.063 | 0.063 | -0.16 | 0.16 |
| Lumbar spine BMD | | GEFOS | 28,498 | 7.42E-03 | -0.038 | -0.038 | -0.071 | -0.068 | 0.068 | -0.17 | 0.17 |
| Heel BMD | | UKB | 142,487 | -4.11E-03 | -0.011 | -0.003 | -0.046 | -0.03 | 0.03 | -0.075 | 0.075 |
| Total body BMD | | GEFOS | 66,628 | -5.52E-04 | -0.083 | 0.069 | -0.030 | -0.045 | 0.045 | -0.11 | 0.11 |
| Total body BMD of age 0-15 | | GEFOS | 11,807 | -1.10E-03 | -0.002 | 0.104 | -0.015 | -0.105 | 0.105 | -0.27 | 0.27 |
| Total body BMD of age 15-30 | | GEFOS | 4180 | -0.010 | -0.145 | 0.056 | -0.032 | -0.18 | 0.18 | -0.49 | 0.49 |
| Total body BMD of age 30-45 | | GEFOS | 10,062 | -1.38E-04 | -0.048 | -0.017 | -0.023 | -0.116 | 0.116 | -0.29 | 0.29 |
| Total body BMD of age 45-60 | | GEFOS | 18,805 | -5.66E-03 | -0.135 | 0.087 | -0.032 | -0.085 | 0.085 | -0.21 | 0.21 |
| Total body BMD of age over 60 | | GEFOS | 22,504 | 6.34E-03 | -0.074 | 0.048 | -0.039 | -0.077 | 0.077 | -0.19 | 0.19 |

Betas were obtained from the random-effects inverse-variance weighted model; NA, not available.

Power was calculated using an online tool: http://cnsgenomics.com/shiny/mRnd/.

**Supplementary Table 5.** Associations of genetic prediction of circulating homocysteine with bone mineral density in different regions and age strata in sensitivity analyses.

| Source | Outcome | SNPs  used | *Q* | *P (Q)* | Weighted median | | | | MR-Egger | | | | | |
| --- | --- | --- | --- | --- | --- | --- | --- | --- | --- | --- | --- | --- | --- | --- |
|  |  |  |  |  | Effect | 95% CI | | *P* | Effect | 95% CI | | *P* | | *P_intercept_* |
| GEFOS | Forearm BMD | 11 | 22.0 | 0.015 | 0.052 | -0.136 | 0.240 | 0.590 | 0.198 | -0.266 | 0.662 | | 0.403 | 0.523 |
| GEFOS | Femoral neck BMD | 9 | 11.6 | 0.169 | -0.029 | -0.143 | 0.086 | 0.621 | -0.148 | -0.479 | 0.183 | | 0.382 | 0.648 |
| GEFOS | Lumbar spine BMD | 9 | 15.7 | 0.047 | -0.089 | -0.229 | 0.050 | 0.209 | 0.181 | -0.233 | 0.596 | | 0.391 | 0.198 |
| UKB | Heel BMD | 11 | 6.2 | 0.801 | -0.045 | -0.081 | -0.010 | 0.013 | -0.035 | -0.098 | 0.027 | | 0.268 | 0.701 |
| GEFOS | Total body BMD | 11 | 10.1 | 0.432 | -0.040 | -0.103 | 0.024 | 0.220 | -0.083 | -0.193 | 0.026 | | 0.137 | 0.285 |
| GEFOS | Total body BMD of age 0-15 | 11 | 9.3 | 0.502 | -0.060 | -0.204 | 0.085 | 0.418 | -0.183 | -0.427 | 0.062 | | 0.142 | 0.134 |
| GEFOS | Total body BMD of age 15-30 | 11 | 10.0 | 0.441 | -0.015 | -0.260 | 0.230 | 0.905 | 0.003 | -0.454 | 0.461 | | 0.990 | 0.871 |
| GEFOS | Total body BMD of age 30-45 | 11 | 6.8 | 0.740 | 0.050 | -0.107 | 0.206 | 0.535 | 0.229 | -0.050 | 0.508 | | 0.108 | 0.047 |
| GEFOS | Total body BMD of age 45-60 | 11 | 7.0 | 0.729 | -0.038 | -0.156 | 0.080 | 0.526 | -0.067 | -0.268 | 0.134 | | 0.512 | 0.707 |
| GEFOS | Total body BMD of age over 60 | 11 | 9.9 | 0.448 | -0.062 | -0.173 | 0.049 | 0.276 | -0.085 | -0.277 | 0.106 | | 0.383 | 0.599 |

BMD, body mineral density; CI, confidence interval; GEFOS, GEnetic Factors for OSteoporosis Consortium; The summary statistics data utilized in this study can be downloaded from the GWAS Catalog (https://www.ebi.ac.uk/gwas/) and Neale Lab (http://www.nealelab.is/uk-biobank); SNP, single nucleotide polymorphism; The Q statistic was used to present the heterogeneity among estimates for each SNPs in one analysis; Effect means the value of Beta; The P value for the intercept in the MR-Egger regression was used to present the pleiotropy (P < 0.05).

**Supplementary Table 6.** Associations of genetic prediction of circulating vitamin B12 with bone mineral density in different body regions and age strata in sensitivity analyses

| Source | Outcome | SNPs  used | *Q* | *P (Q)* | Weighted median | | | | MR-Egger | | | | |
| --- | --- | --- | --- | --- | --- | --- | --- | --- | --- | --- | --- | --- | --- |
|  |  |  |  |  | Effect | 95% CI | | *P* | Effect | 95% CI | | *P* | *P_intercept_* |
| GEFOS | Forearm BMD | 8 | 6.1 | 0.525 | -0.053 | -0.216 | 0.111 | 0.528 | -0.087 | -0.318 | 0.143 | 0.457 | 0.988 |
| GEFOS | Femoral neck BMD | 7 | 7.7 | 0.260 | -0.056 | -0.145 | 0.033 | 0.216 | 0.037 | -0.081 | 0.156 | 0.538 | 0.100 |
| GEFOS | Lumbar spine BMD | 7 | 11.8 | 0.066 | -0.083 | -0.207 | 0.042 | 0.194 | -0.008 | -0.193 | 0.176 | 0.929 | 0.690 |
| UKB | Heel BMD | 11 | 16.0 | 0.100 | 0.005 | -0.018 | 0.029 | 0.659 | 0.009 | -0.023 | 0.041 | 0.564 | 0.089 |
| GEFOS | Total body BMD | 11 | 13.2 | 0.212 | -0.053 | -0.110 | 0.004 | 0.067 | -0.032 | -0.099 | 0.035 | 0.345 | 0.056 |
| GEFOS | Total body BMD of age 0-15 | 11 | 7.5 | 0.677 | -0.043 | -0.159 | 0.073 | 0.466 | 0.140 | -0.013 | 0.293 | 0.073 | 0.025 |
| GEFOS | Total body BMD of age 15-30 | 10 | 10.7 | 0.296 | -0.149 | -0.360 | 0.063 | 0.168 | -0.054 | -0.301 | 0.192 | 0.666 | 0.352 |
| GEFOS | Total body BMD of age 30-45 | 11 | 9.2 | 0.518 | -0.058 | -0.176 | 0.060 | 0.333 | -0.070 | -0.223 | 0.084 | 0.373 | 0.728 |
| GEFOS | Total body BMD of age 45-60 | 11 | 9.8 | 0.456 | -0.115 | -0.210 | -0.020 | 0.018 | -0.082 | -0.192 | 0.028 | 0.142 | 0.222 |
| GEFOS | Total body BMD of age over 60 | 11 | 4.4 | 0.928 | -0.090 | -0.177 | -0.003 | 0.042 | -0.025 | -0.146 | 0.096 | 0.687 | 0.343 |

BMD, body mineral density; CI, confidence interval; GEFOS, GEnetic Factors for OSteoporosis Consortium; The summary statistics data utilized in this study can be downloaded from the GWAS Catalog (https://www.ebi.ac.uk/gwas/) and Neale Lab (http://www.nealelab.is/uk-biobank); SNP, single nucleotide polymorphism; The Q statistic was used to present the heterogeneity among estimates for each SNPs in one analysis; Effect means the value of Beta; The P value for the intercept in the MR-Egger regression was used present the pleiotropy (P < 0.05).

**Supplementary Table 7.** Associations of genetic prediction of circulating homocysteine and vitamin B12 with bone mineral density in different regions and age strata in the MR-PRESSO analysis

| Source | Outcome | | Homocysteine | | | | | | | | | | Vitamin B12 | | | | | | |
| --- | --- | --- | --- | --- | --- | --- | --- | --- | --- | --- | --- | --- | --- | --- | --- | --- | --- | --- | --- |
|  |  |  | SNPs  used | | Outliers | | P_Glo | P_Dis | Effect | SE | | *P* | SNPs  used | Outliers | P_Glo | P_Dis | Effect | SE | *P* |
| GEFOS | | Forearm BMD | 11 | 0 | | 0.024 | | NA | 0.062 | | 0.101 | 0.551 | 8 | 0 | 0.416 | NA | -0.089 | 0.059 | 0.173 |
| GEFOS | | Femoral neck BMD | 9 | 0 | | 0.145 | | NA | -0.075 | | 0.051 | 0.183 | 7 | 0 | 0.29 | NA | -0.043 | 0.041 | 0.330 |
| GEFOS | | Lumbar spine BMD | 9 | 0 | | 0.046 | | NA | -0.077 | | 0.070 | 0.303 | 7 | 0 | 0.156 | NA | -0.038 | 0.053 | 0.499 |
| UKB | | Heel BMD | 11 | 0 | | 0.769 | | NA | -0.046 | | 0.011 | 1.79E-03 | 11 | 0 | 0.148 | NA | -0.012 | 0.011 | 0.323 |
| GEFOS | | Total body BMD | 11 | 0 | | 0.448 | | NA | -0.029 | | 0.024 | 0.257 | 11 | 0 | 0.12 | NA | -0.085 | 0.022 | 3.57E-03 |
| GEFOS | | Total body BMD of age 0-15 | 11 | 0 | | 0.469 | | NA | -0.014 | | 0.053 | 0.793 | 11 | 0 | 0.594 | NA | -0.004 | 0.040 | 0.921 |
| GEFOS | | Total body BMD of age 15-30 | 11 | 0 | | 0.507 | | NA | -0.031 | | 0.097 | 0.757 | 10 | 0 | 0.254 | NA | -0.147 | 0.076 | 0.086 |
| GEFOS | | Total body BMD of age 30-45 | 11 | 0 | | 0.721 | | NA | -0.026 | | 0.052 | 0.632 | 11 | 0 | 0.577 | NA | -0.048 | 0.046 | 0.319 |
| GEFOS | | Total body BMD of age 45-60 | 11 | 0 | | 0.743 | | NA | -0.032 | | 0.038 | 0.408 | 11 | 0 | 0.307 | NA | -0.136 | 0.034 | 2.71E-03 |
| GEFOS | | Total body BMD of age over 60 | 11 | 0 | | 0.445 | | NA | -0.039 | | 0.041 | 0.364 | 11 | 0 | 0.93 | NA | -0.074 | 0.023 | 8.77E-03 |

BMD, body mineral density; GEFOS, GEnetic Factors for OSteoporosis Consortium; The summary statistics data utilized in this study can be downloaded from the GWAS Catalog (https://www.ebi.ac.uk/gwas/) and Neale Lab (http://www.nealelab.is/uk-biobank); SE, standard error; SNP, single nucleotide polymorphism; NA, not available; Effect means the value of Beta; P_Glo, p value for global test; P_Dis, p value for distortion test.

**Supplementary Table 8.** Associations of genetic prediction of B vitamins, homocysteine with fractures in different regions by IVW method.

| Exposure | Outcome | SNPs  used | IVW | | | |
| --- | --- | --- | --- | --- | --- | --- |
|  |  |  | Effect | 95% CI | | *P* |
| Hcy | Forearm fracture | 11 | 6.99E-05 | -1.87E-03 | 2.01E-03 | 0.944 |
|  | Femoral neck fracture | 11 | -8.42E-04 | -2.33E-03 | 6.48E-04 | 0.268 |
|  | Lumbar spine fracture | 9 | 1.09E-03 | -3.93E-06 | 2.19E-03 | 0.051 |
|  | Heel fracture | 11 | 4.07E-04 | -7.88E-04 | 1.60E-03 | 0.505 |
| Vitamin B12 | Forearm fracture | 11 | 5.89E-04 | -6.24E-04 | 1.80E-03 | 0.341 |
|  | Femoral neck fracture | 11 | 1.37E-04 | -7.38E-04 | 1.01E-03 | 0.759 |
|  | Lumbar spine fracture | 4 | 3.39E-04 | -7.60E-04 | 1.44E-03 | 0.546 |
|  | Heel fracture | 11 | -2.31E-04 | -9.77E-04 | 5.16E-04 | 0.545 |
| Folate | Forearm fracture | 2 | 2.94E-04 | -4.31E-03 | 4.90E-03 | 0.900 |
|  | Femoral neck fracture | 2 | 2.44E-03 | -8.27E-04 | 5.71E-03 | 0.143 |
|  | Lumbar spine fracture | 1 | -1.12E-03 | -3.61E-03 | 1.37E-03 | 0.378 |
|  | Heel fracture | 2 | -2.78E-03 | -5.98E-03 | 4.17E-04 | 0.088 |
| Vitamin B6 | Forearm fracture | 1 | 1.43E-04 | -1.85E-04 | 4.71E-04 | 0.392 |
|  | Femoral neck fracture | 1 | -8.38E-05 | -3.14E-04 | 1.47E-04 | 0.476 |
|  | Lumbar spine fracture | 1 | -1.19E-04 | -2.81E-04 | 4.31E-05 | 0.150 |
|  | Heel fracture | 1 | -3.53E-05 | -2.36E-04 | 1.65E-04 | 0.730 |

CI, confidence interval; The summary statistics data utilized for fractures can be downloaded from Neale Lab (http://www.nealelab.is/uk-biobank); SNP, single nucleotide polymorphism; Effect means the value of log transformed odds ratio.

**Supplementary Table 9.** Associations of genetic prediction of vitamin B12, homocysteine with fractures in different regions by MR-Egger and Weighted median estimate.

| Exposure | Outcome | SNPs  used | Weighted median | | | | MR-Egger | | | | |
| --- | --- | --- | --- | --- | --- | --- | --- | --- | --- | --- | --- |
|  |  |  | Effect | 95% CI | | *P* | Effect | 95% CI | | *P* | *P_intercept_* |
| Hcy | Forearm fracture | 11 | -2.70E-04 | -2.95E-03 | 2.41E-03 | 0.843 | 2.27E-04 | -4.55E-03 | 5.01E-03 | 0.926 | 0.945 |
|  | Femoral neck fracture | 11 | -1.34E-03 | -3.20E-03 | 5.16E-04 | 0.157 | -1.19E-03 | -4.85E-03 | 2.48E-03 | 0.525 | 0.835 |
|  | Lumbar spine fracture | 9 | 9.48E-04 | -4.10E-04 | 2.31E-03 | 0.171 | 1.67E-03 | -9.86E-04 | 4.32E-03 | 0.218 | 0.649 |
|  | Heel fracture | 11 | 8.91E-04 | -6.46E-04 | 2.43E-03 | 0.256 | 1.46E-03 | -1.29E-03 | 4.21E-03 | 0.297 | 0.405 |
| Vitamin B12 | Forearm fracture | 11 | 1.67E-04 | -1.35E-03 | 1.68E-03 | 0.829 | 7.23E-05 | -1.80E-03 | 1.95E-03 | 0.940 | 0.472 |
|  | Femoral neck fracture | 11 | 2.68E-05 | -1.09E-03 | 1.14E-03 | 0.962 | 1.41E-04 | -1.22E-03 | 1.50E-03 | 0.839 | 0.998 |
|  | Lumbar spine fracture | 4 | 2.22E-04 | -9.77E-04 | 1.42E-03 | 0.717 | -3.47E-04 | -2.90E-03 | 2.20E-03 | 0.790 | 0.559 |
|  | Heel fracture | 11 | -3.77E-04 | -1.30E-03 | 5.43E-04 | 0.422 | -6.79E-04 | -1.83E-03 | 4.74E-04 | 0.248 | 0.309 |

CI, confidence interval; The summary statistics data utilized for fractures can be downloaded from Neale Lab (http://www.nealelab.is/uk-biobank); SNP, single nucleotide polymorphism; Effect means the value of log transformed odds ratio; The P value for the intercept in the MR-Egger regression was used present the pleiotropy (P < 0.05).

**Supplementary Table 10.** Associations of genetic prediction of vitamin B12, homocysteine with fractures in different regions by MR-PRESSO analysis

| Exposure | Outcome | SNPs  used | Outliers | | P_Glo | P_Dis | | | Effect | SE | *P* |
| --- | --- | --- | --- | --- | --- | --- | --- | --- | --- | --- | --- |
| Hcy | Forearm fracture | 11 | 0 | 0.427 | | | NA | 7.53E-05 | | 1.00E-03 | 0.942 |
|  | Femoral neck fracture | 11 | 0 | 0.281 | | | NA | -8.37E-04 | | 7.70E-04 | 0.303 |
|  | Lumbar spine fracture | 9 | 0 | 0.254 | | | NA | 1.11E-03 | | 5.63E-04 | 0.083 |
|  | Heel fracture | 11 | 0 | 0.671 | | | NA | 4.10E-04 | | 5.30E-04 | 0.458 |
| Vitamin B12 | Forearm fracture | 11 | 0 | 0.834 | | | NA | 5.97E-04 | | 4.68E-04 | 0.231 |
|  | Femoral neck fracture | 11 | 0 | 0.684 | | | NA | 1.40E-04 | | 3.89E-04 | 0.727 |
|  | Lumbar spine fracture | 4 | 0 | 0.94 | | | NA | 3.39E-04 | | 2.22E-04 | 0.224 |
|  | Heel fracture | 11 | 0 | 0.642 | | | NA | -2.23E-04 | | 3.55E-04 | 0.544 |

The summary statistics data utilized for fractures can be downloaded from Neale Lab (http://www.nealelab.is/uk-biobank); SE, standard error; SNP, single nucleotide polymorphism; NA, not available; Effect means the value of log transformed odds ratio; P_Glo, p value for global test; P_Dis, p value for distortion test.

**Supplementary Table 11.** Associations of genetic prediction of vitamin B12, homocysteine with femoral neck BMD in men by various MR analyses

| Exposure - Outcome Source | Method | Effect | SE | *P* |
| --- | --- | --- | --- | --- |
| Homocysteine - femoral neck BMD in men from GEFOS Consortium | IVW-multiplicative random effects | -0.045 | 0.085 | 0.598 |
|  | Weighted median estimate | 0.022 | 0.081 | 0.789 |
|  | MR-PRESSO | -0.045 | 0.085 | 0.611 |
|  | Cochrane's Q = 15.64 (*P* = 0.080); MR-Egger intercept = -0.027 (*P* = 0.070) | | | |
| Vitamin B12 - femoral neck BMD in men from GEFOS Consortium | IVW-multiplicative random effects | -0.103 | 0.064 | 0.111 |
|  | Weighted median estimate | -0.095 | 0.075 | 0.207 |
|  | MR-PRESSO | -0.103 | 0.053 | 0.126 |
|  | Cochrane's Q = 2.73 (*P* = 0.604); MR-Egger intercept = -0.004 (*P* = 0.813) | | | |

BMD, bone mineral density; GEFOS, the GEnetic Factors for Osteoporosis (GEFOS) consortium; SE, standard error; IVW, inverse variance weighted; MR, Mendelian randomization; MR-PRESSO, Mendelian randomization pleiotropy residual sum and outlier.

**Supplementary Table 12.** Associations of genetic prediction of vitamin B12, homocysteine with femoral neck BMD in women by various MR analyses

| Exposure - Outcome Source | Method | Effect | SE | *P* |
| --- | --- | --- | --- | --- |
| Homocysteine - femoral neck BMD in women from GEFOS Consortium | IVW-multiplicative random effects | -0.053 | 0.046 | 0.247 |
|  | Weighted median estimate | -0.067 | 0.059 | 0.254 |
|  | MR-PRESSO | -0.053 | 0.034 | 0.146 |
|  | Cochrane's Q = 4.76 (*P* = 0.854); MR-Egger intercept = 0.006 (*P* = 0.317) | | | |
| Vitamin B12 - femoral neck BMD in women from GEFOS Consortium | IVW-multiplicative random effects | -0.092 | 0.066 | 0.166 |
|  | Weighted median estimate | -0.084 | 0.067 | 0.212 |
|  | MR-PRESSO | -0.092 | 0.066 | 0.238 |
|  | Cochrane's Q = 8.32 (*P* = 0.081); MR-Egger intercept = -0.011 (*P* = 0.626) | | | |

BMD, bone mineral density; GEFOS, the GEnetic Factors for Osteoporosis (GEFOS) consortium; SE, standard error; IVW, inverse variance weighted; MR, Mendelian randomization; MR-PRESSO, Mendelian randomization pleiotropy residual sum and outlier.

**Supplementary Table 13.** Associations of genetic prediction of vitamin B12, homocysteine with lumbar spine BMD in men by various MR analyses

| Exposure - Outcome Source | Method | Effect | SE | *P* |
| --- | --- | --- | --- | --- |
| Homocysteine - lumbar spine BMD in men from GEFOS Consortium | IVW-multiplicative random effects | -0.064 | 0.066 | 0.328 |
|  | Weighted median estimate | -0.046 | 0.087 | 0.594 |
|  | MR-PRESSO | -0.064 | 0.058 | 0.298 |
|  | Cochrane's Q = 7.07 (*P* = 0.630); MR-Egger intercept = -0.006 (*P* = 0.566) | | | |
| Vitamin B12 - lumbar spine BMD in men from GEFOS Consortium | IVW-multiplicative random effects | 0.051 | 0.066 | 0.434 |
|  | Weighted median estimate | 0.066 | 0.077 | 0.392 |
|  | MR-PRESSO | 0.051 | 0.051 | 0.369 |
|  | Cochrane's Q = 2.39 (*P* = 0.665); MR-Egger intercept = -0.003 (*P* = 0.859) | | | |

BMD, bone mineral density; GEFOS, the GEnetic Factors for Osteoporosis (GEFOS) consortium; SE, standard error; IVW, inverse variance weighted; MR, Mendelian randomization; MR-PRESSO, Mendelian randomization pleiotropy residual sum and outlier.

**Supplementary Table 14.** Associations of genetic prediction of vitamin B12, homocysteine with lumbar spine BMD in women by various MR analyses

| Exposure - Outcome Source | Method | Effect | SE | *P* |
| --- | --- | --- | --- | --- |
| Homocysteine - lumbar spine BMD in women from GEFOS Consortium | IVW-multiplicative random effects | -0.014 | 0.045 | 0.746 |
|  | Weighted median estimate | -0.046 | 0.056 | 0.418 |
|  | MR-PRESSO | -0.014 | 0.045 | 0.754 |
|  | Cochrane's Q = 9.24 (*P* = 0.416); MR-Egger intercept = 0.007 (*P* = 0.403) | | | |
| Vitamin B12 - lumbar spine BMD in women from GEFOS Consortium | IVW-multiplicative random effects | -0.080 | 0.107 | 0.459 |
|  | Weighted median estimate | -0.152 | 0.070 | 0.031 |
|  | MR-PRESSO | -0.080 | 0.107 | 0.500 |
|  | Cochrane's Q = 3.22 (*P* = 0.573); MR-Egger intercept = -0.024 (*P* = 0.459) | | | |

BMD, bone mineral density; GEFOS, the GEnetic Factors for Osteoporosis (GEFOS) consortium; SE, standard error; IVW, inverse variance weighted; MR, Mendelian randomization; MR-PRESSO, Mendelian randomization pleiotropy residual sum and outlier.

**Supplementary Figure 1.** Effects of genetic prediction of circulating vitamin B12 and homocysteine on BMD in different body regions and different age strata with the Multivariable Mendelian randomization adjusting education attained, smoking and alcohol usage. BMD, body mineral density; CI, confidence interval; GEFOS, GEnetic Factors for OSteoporosis Consortium; The summary statistics data utilized in this study can be downloaded from the GWAS Catalog (https://www.ebi.ac.uk/gwas/) and Neale Lab (http://www.nealelab.is/uk-biobank); *P value reached the significant level; SNP, single nucleotide polymorphism


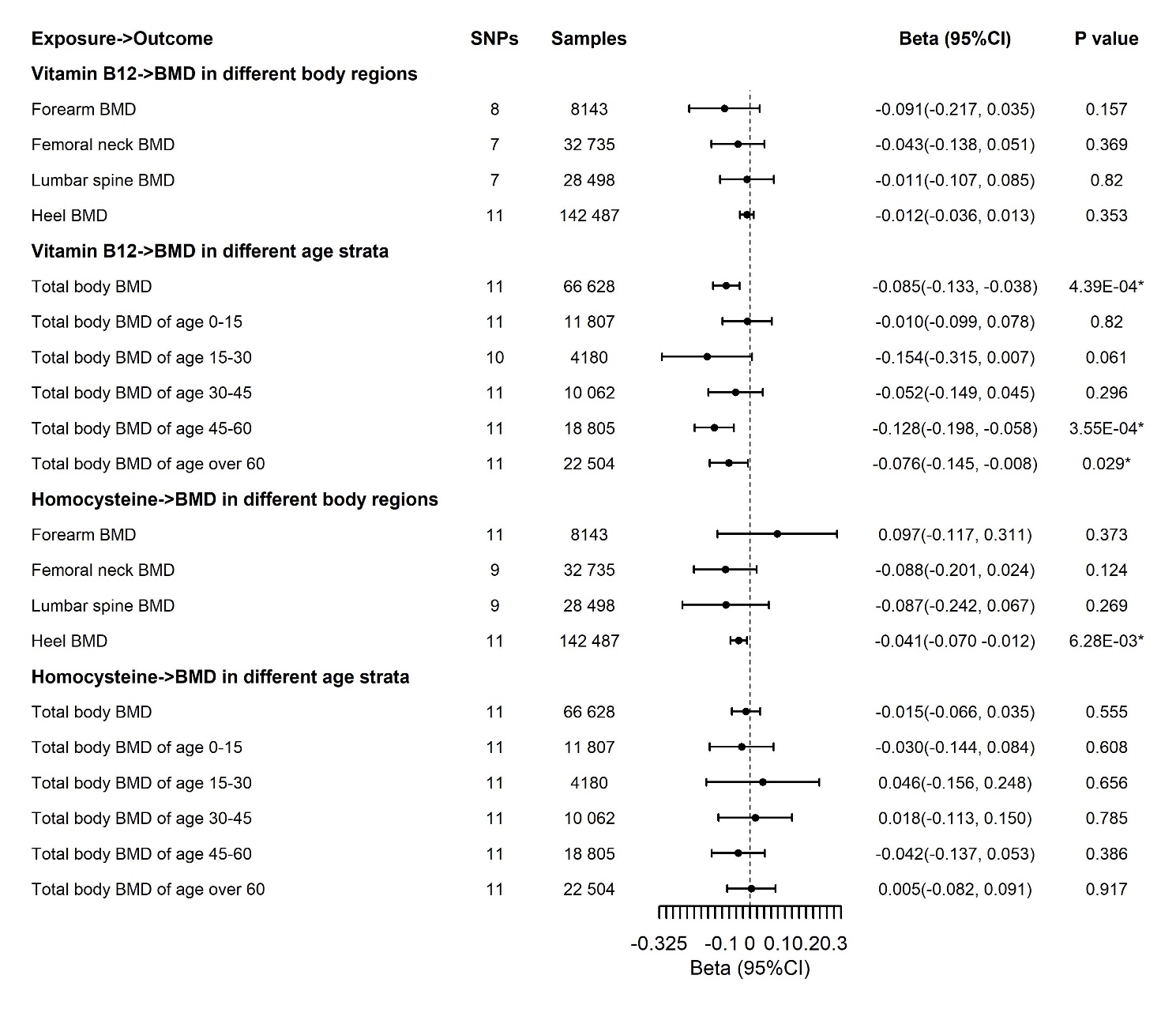


**Supplementary Figure 2.** Forest plot for leave-one-out analysis of the significant IVW results of homocysteine with heel BMD, Vitamin B12 with total body BMD, total body BMD of age 45-60 and age over 60, with each point denoting the causal effect by IVW after removing the specific SNP on the left side. IVW, inverse variance weighted method.


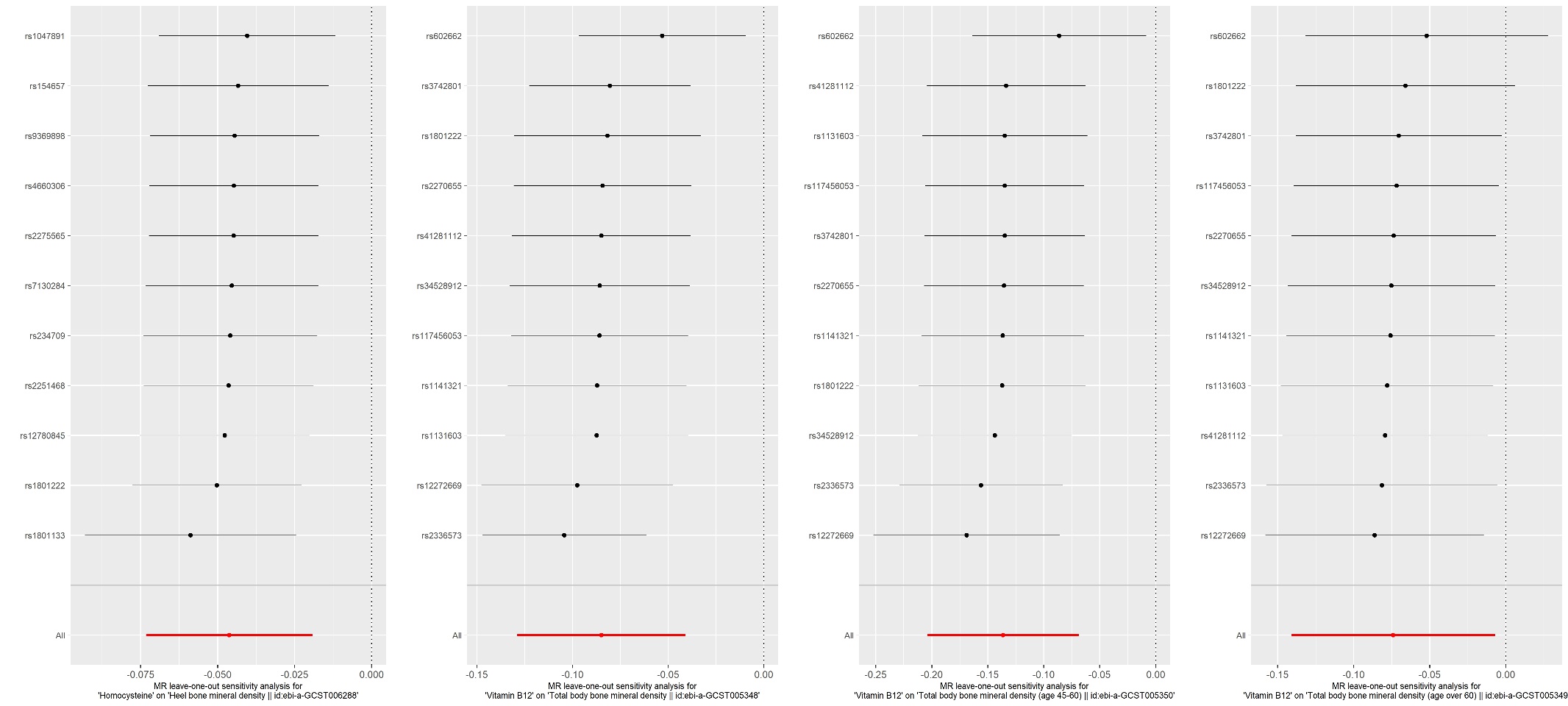

Supplement: Supplementary file 1 [file DataSheet_1.docx]
